# Contrast-Induced Neurotoxicity: An Inside Look at a Rare Presentation

**DOI:** 10.7759/cureus.29627

**Published:** 2022-09-26

**Authors:** Grant E Gregory, Kavan Thompson, James Case, Yogesh Gujrati

**Affiliations:** 1 Medicine, Alabama College of Osteopathic Medicine, Dothan, USA; 2 Internal Medicine, Southeast Health Medical Center, Dothan, USA; 3 Neurology, Southeast Health Medical Center, Dothan, USA

**Keywords:** cerebral vasculopathy, neuro radiology, neuro-imaging, ct (computed tomography) imaging, nuclear magnetic resonance

## Abstract

Imaging modalities frequently utilize iodinated-based contrast agents (IBCAs) to assist professionals in deficit identification and improve clinical outcomes for patients. However, they are not without risk. In patients with post-radiological neurological deficits, contrast-induced neurotoxicity (CIN) should be among the top differentials. In this case report, we present the case of a 61-year-old female who experienced classical signs and symptoms of neurotoxicity after a cerebral angiogram. The patient’s clinical detriments stemming from iodinated contrast resolved after a multi-day treatment of high-dose steroid use.

## Introduction

Iodinated-based contrast agents (IBCAs) are widely used in a range of imaging modalities to improve anatomic identification and to better assess the structural integrity and function of an organ. Often medically necessary and although generally tolerated among patient populations, immediate adverse effects of IBCAs are important to recognize and treat appropriately. IBCA adverse effects are categorized into two distinct categories: allergic-like, and physiologic [1]. Allergic-like reactions include anaphylactic symptoms such as urticaria, dyspnea, nausea, and hypotension [2]. Physiologic (toxic) reactions are dose-dependent and include the well-known adverse effect of nephropathy, along with neurotoxicity and cardiovascular compromise [2,3]. 

Contrast-induced neurotoxicity (CIN) is a rare adverse effect of iodinated-based agents, resulting in temporary to permanent neurological deficits. Proposed mechanisms of CIN include disruption of the blood-brain barrier (BBB) by the high osmolarity of IBCAs, and the subsequent homeostatic imbalance that such an imbalance entails [4]. The resulting cerebral edema complicates recovery efforts in patients afflicted with the rare, but often severe neurotoxic profile of IBCAs [5]. CIN occurs in approximately 3.6% of cerebral angiograms utilizing IBCAs. This article highlights a suspected case and successful treatment of CIN in a 61-year-old female following a cerebral angiogram who experienced focal neurological deficits, headaches, and concomitant psychosis [6].

## Case presentation

A 60-year-old Caucasian female with hypertension, type-II diabetes mellitus, and hyperlipidemia was diagnosed and treated for a right-hemispheric stroke in early 2019. Later that year, the patient subsequently suffered a left-hemispheric stroke and was treated. Incidentally at this time, an unruptured anterior communicating artery (ACA) aneurysm was identified on neuroimaging as well as lesions concerning for right and left-sided internal carotid artery (ICA) aneurysms. Primary coil embolization took place in the following months to eradicate the well-established ACA aneurysm evidenced by neuroimaging. Following the procedure, the patient moved across state lines, and primary care was established in the new location. A full history and physical was conducted upon initial primary care evaluation, which was remarkable for 80% strength in all four extremities and decreased sensation to light touch in all four extremities. The patient also complained of frequent headaches and visual disturbances. 

Pertinent to the history of the present illness, the patient was previously diagnosed with anxiety, neuropathy, and overflow incontinence. Her medications included alprazolam 0.5 mg qid, amlodipine 10 mg qid, atorvastatin 20 mg qid, buspirone 5 mg bid, clopidogrel 75 mg qid, duloxetine 30 mg qid, gabapentin 800 mg bid, 20 units of insulin qid, oxybutynin 10 mg qid, and propranolol 40 mg qid. Her prior surgical history includes an appendectomy, a cardiac pacemaker placement, a cesarean section delivery, a cholecystectomy, and a hysterectomy. In addition, her social history was significant for a 10-pack-year history of smoking. 

Due to the uncertainty surrounding her history of cerebrovascular compromise and aneurysm identification at an outside facility, and in conjunction with her current symptoms, she was referred to a neurovascular specialist for a follow-up.

Upon the initial neurovascular consultation, baseline magnetic resonance imaging (MRI) images were obtained. MRI imaging demonstrated encephalomalacia in the subcortical and cortical distribution of the right hemisphere. There was no evidence of a residual aneurysm in the ACA. A cerebral angiogram was performed to further assess intracerebral and extracranial circulation, in addition to evaluating for the possibility of any residual aneurysms in the ACA and ICAs. The angiogram revealed bilateral intracranial ICA atherosclerotic disease without a cerebral aneurysm.

Post-procedurally, the patient developed an altered mental status with an inability to answer basic questions, lethargy, and diffuse left-sided body weakness in the upper and lower extremities on physical exam. The patient also developed slurred speech. The patient complained of intense pressure behind her right eye and experienced nausea and non-bloody vomitus.

**Figure 1:**
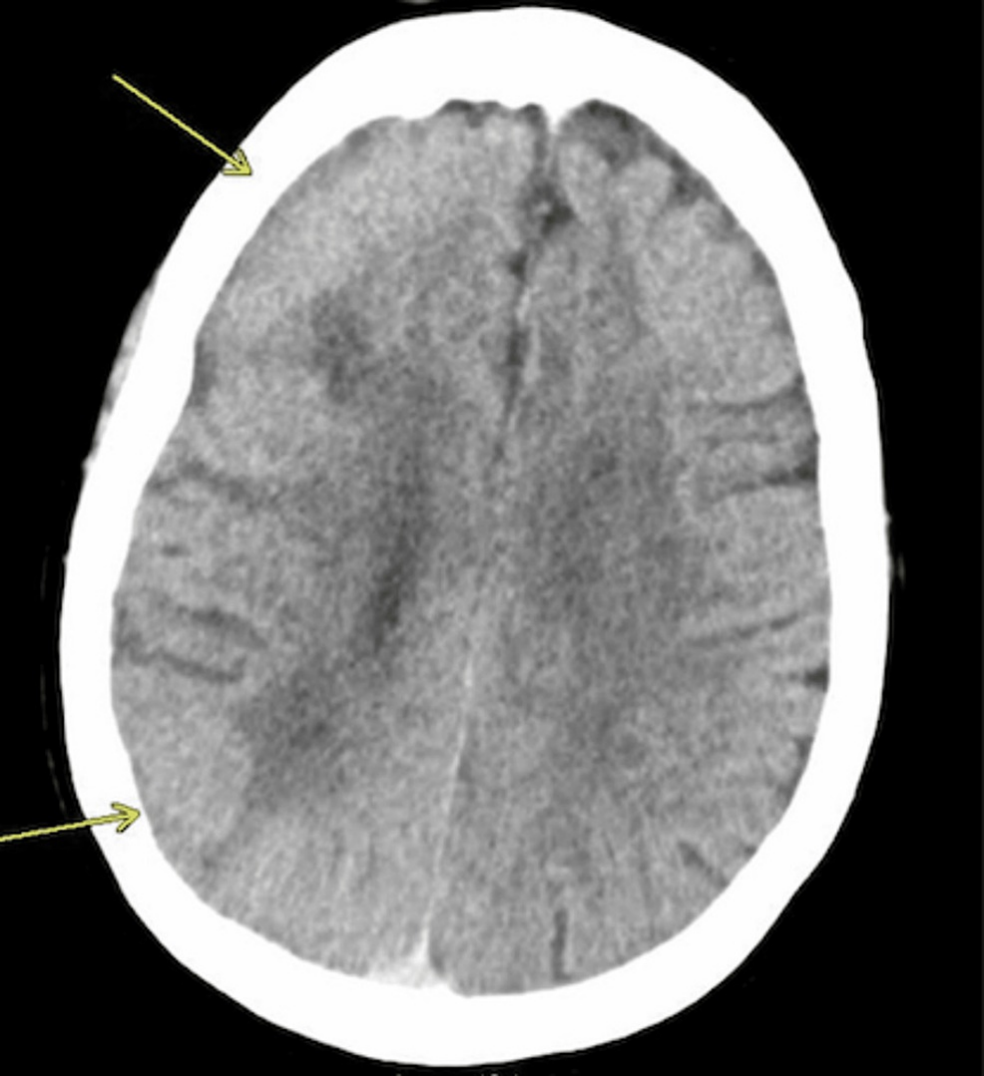
CT scan of the head in the immediate perioperative period after dedicated catheter angiogram study. Asymmetric effacement of cerebrospinal fluid spaces and subtle loss of the gray-white matter differentiation along the right cerebral convexity could represent acute or subacute infarction. An MRI is the best test to confirm.

**Figure 2:**
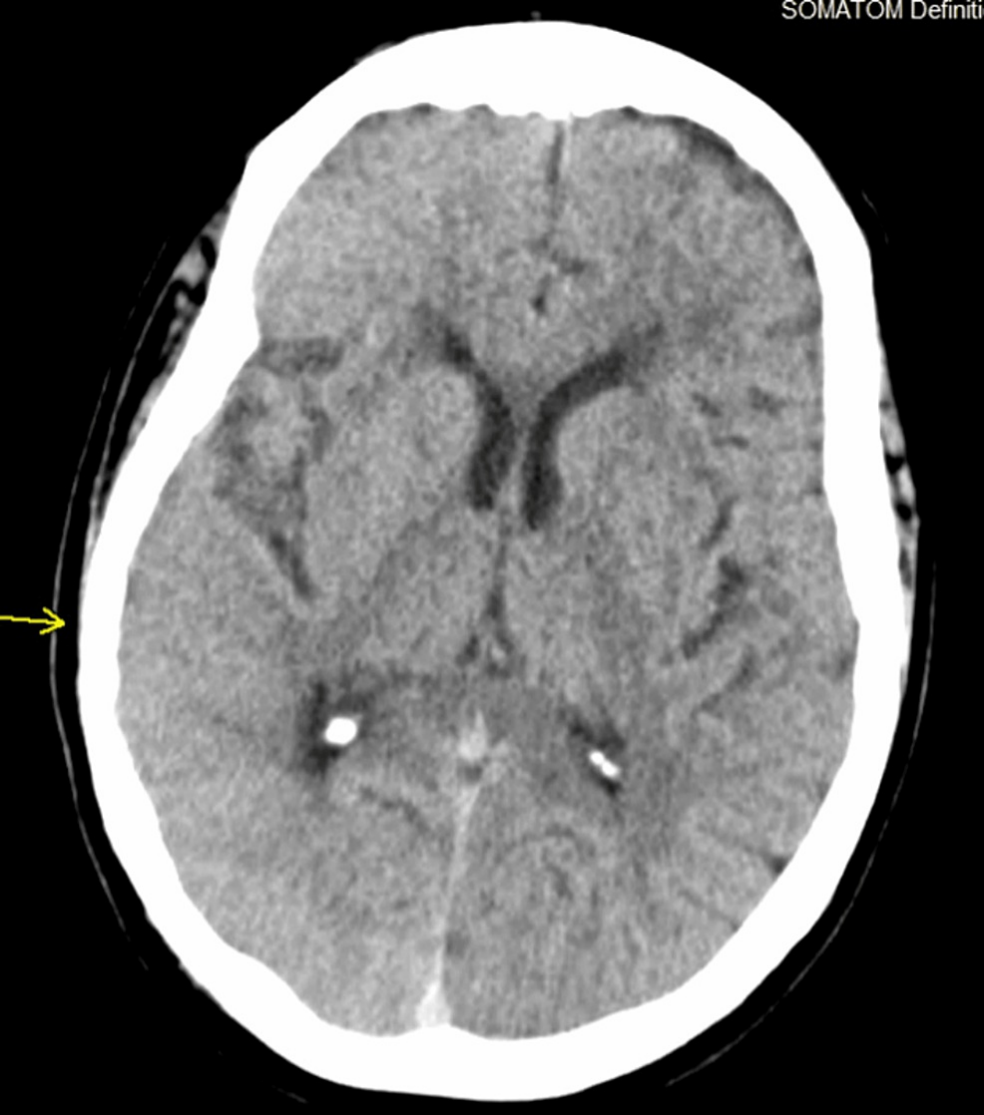
CT scan of the head in the immediate perioperative period after dedicated catheter angiogram study.

**Figure 3:**
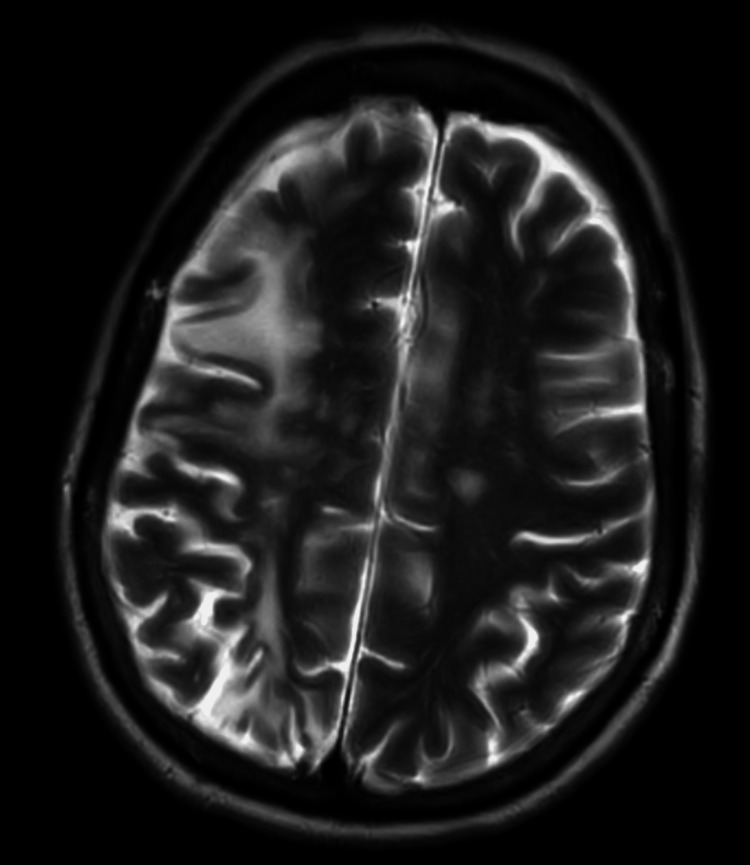
Fluid-attenuated inversion recovery (FLAIR) MRI in the immediate perioperative period. Changes of an old right MCA territory infarction with areas of gliosis and subcortical white matter hyperintensity. Chronic ischemic changes are noted. No evidence of acute intracranial abnormality.

The patient was subsequently admitted for in-patient care. Her vital signs on admission showed a blood pressure (BP) of 148/65, heart rate (HR) of 80, respiratory rate (RR) of 16, and a 94% oxygen saturation reading on room air. She was mildly febrile with a temperature of 100.4 Fahrenheit. An electrocardiogram (ECG) demonstrated normal sinus rhythm. A chest X-ray (CXR) revealed no acute cardiopulmonary abnormality. A complete metabolic profile returned and showed labs within normal limits. Computed tomography (CT) neuroimaging revealed sulcal effacement and loss of gray-white matter differentiation in the right cerebral hemisphere, as depicted in Figures 1-2. An MRI was ordered to confirm the absence of an acute infarct as depicted in Figure 3. At this time, the relationship between the administration of intravenous contrast and the development of symptoms was consistent with CIN.

The patient was started on 250 mg of IV methylprednisolone qid and IV levetiracetam 750 mg bid for seizure prophylaxis, in addition to ensuring adequate hydration with normal saline. The patient was also started on IV ceftriaxone 1g q.d. for a concomitant urinary tract infection, as demonstrated on urinalysis upon admission. 

**Figure 4:**
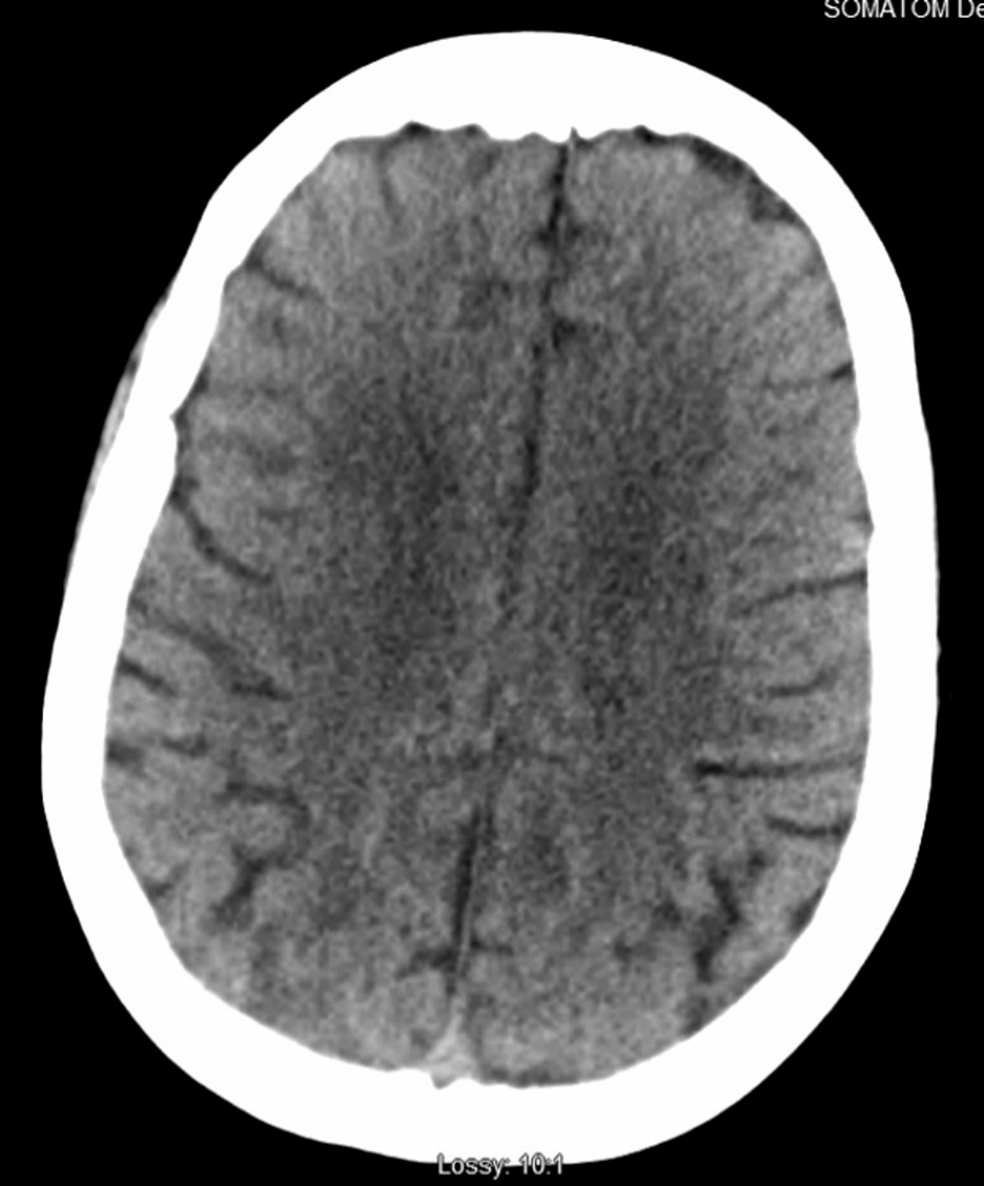
CT scan of the head 24 hours later after treatment with IV corticosteroids. Significantly improved gray-white matter differentiation. No acute intracranial abnormality is noted.

One day later, the patient’s neuroimaging had improved with a CT scan demonstrating normal restoration of gray-white matter differentiation in the right cerebral hemisphere as depicted in Figure 4. However, she had not improved functionally and continued to complain of a right-sided headache. In addition, she began to experience hallucinations and became combative towards her daughter and the hospital staff, and a psychiatry consult was requested. After a psychiatric evaluation, the acute psychosis was believed to be multifactorial and related to the contrast angiogram in addition to recent high-dose steroid use and the concomitant urinary tract infection. Serum creatinine and electrolytes were still within normal limits at this time. The plan was agreed upon by the hospitalist team to continue methylprednisolone 250 mg qid and continue to reassess for improvements in her clinical condition. 

On examination the next day, the patient's functional neurological status still had not improved to the level of perceived improvement on neuroimaging. The patient demonstrated continual left-sided weakness on physical exam and continued to complain of a right-sided headache. Her psychiatric status still had not improved either, as she remained combative and hallucinatory. An EEG was subsequently performed, which did not reveal any epileptiform abnormalities. The patient care plan, including methylprednisolone 250 mg qid, was to be continued at this time. 

One day later and three days post-steroid initiation, the patient began to improve clinically and regain neurological function, including improvements in both her acute psychotic state and left-extremity motor strength. The patient’s right-sided headache had also begun to dissipate. Laboratory results were normal, which included an apparent resolution of the patient’s UTI. The hospital team made plans to taper methylprednisolone in hope of a discharge in the following days. The IV ceftriaxone and IV levetiracetam regimens were also discontinued at this time.

Five days post steroid initiation and on the morning of discharge, a final neurological exam was performed which revealed intact cranial nerves II-XII, along with intact sensation and ⅘ muscle strength bilaterally in the upper and lower extremities. Laboratory results were unremarkable. The patient’s psychiatric state was notable for significant improvements, as the patient was pleasant, fully oriented, smiling, and answering questions in full sentences. She reported that her headache had fully dissipated. The patient was discharged to an in-patient rehabilitation facility and will be followed in the clinic going forward. 

## Discussion

The use of iodinated contrast agents has become a mainstay in diagnostic radiology, interventional radiology, and interventional cardiology. While the benefit-to-risk profile of using iodinated contrast strongly favors the benefit in most clinical scenarios, using contrast does not come without risk. There are guidelines in place for the use of contrast agents in patients with chronic kidney disease, pregnancy, patients on metformin with decreased kidney function, and patients who have had a previous hypersensitivity reaction to iodinated contrast agents. The common misconception about patients having a hypersensitivity reaction to contrast agents is that the patients will often believe iodine is the cause of the reaction, but iodine is a naturally present element and is not antigenic in itself [7]. The problem with antigenicity arises from the other chemical moieties within the contrast agent. These chemical compounds are constructed to maintain the iodine benefits while promoting hydrophilicity and low osmolality. To increase the chemical’s hydrophilicity, the iodine is usually attached to a benzene ring and hydroxyl groups are added to the other carbons of the benzene ring. There are numerous manufacturers of iodinated contrast and each has its own compounds to maintain its proprietary value. 

The contrast used in our patient report was Omnipaque 350 (Nycomed Amersham, Princeton, NJ, USA). This is considered a lower osmolality and non-ionic contrast while there is a higher risk with high osmolality and ionic compounds. Therefore, Omnipaque 350 should have a lower incidence of CIN but not zero. This highlights that although the properties of the contrast do impact the risk of developing CIN, this case study suggests that those factors are the only ones involved in the pathophysiology. 

A well-documented risk of injection of iodinated contrast has been contrast-induced nephropathy. Contrast-induced nephropathy has been a topic of discussion in the medical community because it does not seem to affect patients clinically unless the patient has certain risk factors. According to an observational study, guidelines were created to decrease the risk of contrast-induced nephropathy, they are as follows: patients with estimated glomerular filtration rates (eGFRs) ≥ 45mL/min/1.73m2 are at negligible risk, while patients with eGFRs<30mL/min/1.73m2 are at high risk for contrast-induced nephropathy. Patients with eGFRs between 30 and 44mL/min/1.73m2 are at an intermediate risk unless diabetes mellitus is present, which would further increase the risk [8]. Although this is not the purpose of the study, there appears to be a relationship between the pathogenesis of contrast-induced nephropathy and CIN. Although the pathogenesis of each is not fully understood, there seems to be a relationship that cannot be ignored, they are both usually transient with rapid recovery. Contrast-induced nephropathy shows signs and symptoms similar to that of acute tubular necrosis (ATN) but with rapid recovery (a few days) unlike other causes of ATN with a slower recovery (one to three weeks). The difference could be explained by a functional change occurring in the tubular cells of the kidney occurring from contrast as opposed to the necrosis of tubular cells seen in other causes of ATN. The transient nature of each contrast-induced nephropathy and CIN suggests that although there are changes occurring at the cellular level, they seem to be reversible although further studies must be done to prove the correlation between the two clinical diagnoses. 

Contrast agents can have both excitatory and inhibitory influences on neurons. The BBB being the gatekeeper of substances to access the parenchyma of the brain is formed from three cell types: endothelial cells of the blood vessel, pericytes surrounding the blood vessels, and astrocyte foot processes. There appears to be greater disruption of the BBB with contrast agents as compared with intravenous mannitol of equivalent osmolarity, thus demonstrating that there are intrinsic properties of contrast agents which contribute to this disruption apart from hyperosmolarity [9]. Areas of the brain which are not protected by the BBB such as certain regions in the hypothalamus and the area postrema may be more susceptible to the effects of contrast agents from exposure to higher concentrations in the blood. The extent of disruption also appears to be related to the duration of the injection. It is important to remember that disease states like certain infections increase the permeability of the BBB and in such states, contrast agents enter the brain more easily, predisposing to further injury. While not well studied, these principles suggest that a patient with encephalomalacia from a previous cerebrovascular accident would have a higher risk for BBB disruption [10]. Glial cells are highly involved in the pathogenesis of encephalomalacia and proliferate in the area of infarction and replace dead tissue. The astrocytes that were previously helping to form the BBB at that site, will no longer contribute to the BBB and strongly decrease the function of the BBB which is formed from three cell types: endothelial cells of the blood vessel, pericytes surrounding the blood vessels, and astrocyte foot processes. Hyperosmolarity of certain contrast agents can disrupt the BBB by drawing water out of brain capillary endothelial cells, causing shrinkage of cells and separation of tight junctions. This separation of tight junctions is compounded by increased intraluminal tension that occurs due to the vasodilatory effects of the iodinated contrast agents and also from the high pressure of the contrast injection. The Starling equation tells us that the permeability of any membrane can be altered by intraluminal osmolarity difference from interstitium osmolarity, intraluminal pressure difference from interstitial space, and the filtration coefficient. All three of these factors are hypothesized to be altered when intravenous contrast agents are given. The intraluminal osmolarity is increased from the agent itself, the intraluminal pressure is increased from the vasodilatory effects of the contrast and the high pressure of the contrast injection. The filtration coefficient is altered by the BBB changes that were elicited previously. 

These changes occur in a rapid manner and in most cases are normalized in a rapid manner as well. The importance of the early recognition of CIN cannot be overstated, as efficient and proper management of symptoms is associated with greater prognoses. Previous analysis of literature in EMBASE and MEDLINE databases identified 48 cases of CIN as of 2020. Of these cases, 60.4% of patients were female and the most common comorbidities included hypertension (60.4%), diabetes (12.5%), and renal impairment (10.5%); 25% of patients had previously had a neuroendovascular procedure using iodinated contrast before developing CIN on their most recent [11]. Symptom analysis identified encephalopathy (39.6%), cortical blindness (39.6%), unilateral motor deficits (37.5%), decreased vigilance (20.8%), headache (18.8%), and aphasia (18.8%) as the most common [11].

Although symptom control remains the primary treatment modality, corticosteroids appear to be the most commonly-used therapeutic in the treatment of CIN (>50%), followed by vigorous hydration, mannitol to decrease cerebral pressure, anticonvulsants for seizure prophylaxis, and respiratory assistance if the airway is compromised; 90% of patients in this series experienced complete recovery at a median recovery time of three days, and one patient expired [11].

Further studies are required prior to any recommendations on which patients are most at risk for CIN. Until the pathogenesis of the finding is further understood in animal models and related to the clinical data that has been compiled, it will be difficult to determine the link to CIN. With that being said, the risk of developing neurotoxicity from iodinated contrast is minimal and there is a high success rate with treatment. Therefore, patient education about the risk and benefits of contrast administration should be discussed with patients on an individual basis and be a collaborative decision between the patient and physician. 

## Conclusions

IBCAs are medically necessary, but not without risk. Increased awareness may prompt faster recognition and more effective treatment of encephalopathic symptoms. Additional research is needed to determine if IBCA toxicity is dose-dependent or idiopathic, and to investigate the best practices associated with management and prevention.
